# Regarding “Willingness to Pay for HIV Prevention Commodities Among Key Population Groups in Nigeria”

**DOI:** 10.9745/GHSP-D-22-00445

**Published:** 2023-02-28

**Authors:** Victor Abiola Adepoju, Kristina Grabbe, Catey Laube, Chidinma Umebido, Zahariyya Hassan, Adetiloye Oniyire

**Affiliations:** a Jhpiego, Abuja, Nigeria.; b Jhpiego, Baltimore, MD, USA.

See related article by Durosinmi-Etti et al.

The study by Durosinmi-Etti et al.^1^ explored the willingness to pay (WTP) for HIV prevention commodities (i.e., HIV self-testing [HIVST], preexposure prophylaxis, and condoms) among 1,169 individuals identifying as key populations (KPs), which consisted of female sex workers and men who have sex with men (MSM) in 3 Nigerian states. The authors reported that 81% of the respondents were willing to pay for HIVST, 32% were willing to pay the retail market price of Nigerian Naira (N)1,600 (US$4.20), and the majority (42%) of KPs were willing to pay lower than the market price (i.e., N500–N1,500 (US$1.30–US$4.00). However, the study recognized certain limitations. The authors recommended integrating supply- and demand-side interventions, such as cross-subsidizing prevention commodities through government insurance, subsidy, the Basic Health Care Provision Fund, a tax waiver for imported commodities, and social marketing to accommodate the various market segments comprising a spectrum of incomes. This study has further advanced our understanding of self-reported WTP for these lifesaving HIV prevention tools and provided new insights that could guide HIV prevention policy development and program planning for KPs in Nigeria.

Despite the laudable findings of this study, there are gaps and limitations that future studies need to address. First, the study did not address how WTP affects future purchasing and frequency of purchasing given that not all respondents may be willing to pay N1,600 (US$4.20) every 3 months (i.e., if experiencing frequent potential HIV exposures and/or retesting for preexposure prophylaxis). Initial or first-time WTP may not translate into WTP as often as the commodity is needed. One of the goals of HIVST is to achieve social and behavioral change and demand for future use.^2^ Therefore, HIVST may be of utility by increasing initial and repeat testing.^3^ For instance, 67.4% of Australian MSM would test more frequently if the test is convenient: self-administered with an immediate result.^4^ However, in South Africa, high cost was highlighted as a major barrier to repeat HIVST following initial exposure to HIVST, especially among high sexual risk-taking populations, a possible behavioral response to risk exposure.^5^ Therefore, the feasibility of HIVST for repeat testing and the impact of cost on WTP for repeat or future HIVST are gaps in implementation science research that HIV prevention initiatives must urgently address.

Moreover, rather than reporting on observed demand for HIVST, the Durosinmi-Etti et al. study explored self-reported WTP for HIVST among KPs. Self-reported WTP studies are largely prone to exaggerated responses compared to actual demand. Chang et al. conducted a randomized controlled trial in Zimbabwe to explore how price changes impact demand for HIVST among 4,000 adults. The authors observed a sharp and significant decline in demand for HIVST, from 32.5% when the commodity was distributed free of charge to less than 3% when the price was N443 (US$1.00) and more. Their finding further strengthens the argument that stated WTP studies are usually exaggerated when compared to observed/actual WTP in real-world programming where low-income populations could be highly sensitive to price increases and demand for prevention products.^6^

Furthermore, the study failed to adjust for the effect of global economic crisis, inflation, dip in external reserves, instability of local currency, and forex scarcity that have mostly affected low- and middle-income countries. These economic indices have had a disproportionate and wider impact on final commodity cost to the end user and WTP but were not considered in the WTP estimation for the study. A similar study conducted in 2018 by Tun et al. reported a WTP of US$5.50 among MSM in Nigeria, equivalent to N2,000 when the official exchange rate was N364 to US$1. By 2022, a study in the same setting and population by Durosinmi-Etti et al. reported a WTP of N1,600, the equivalent of US$4.20 when the exchange rate was N381 for US$1.^1^^,^^7^ It is difficult to compare these studies without standardizing with the current exchange rate, which gives a WTP of US$4.20 in 2022 and US$5.20 in 2018. Due to forex scarcity, many local distributors cannot get forex at the official rate from the central bank and have been forced to buy forex in the more expensive “black market” to settle commodity payment for manufacturers, which are usually paid in US dollars rather than the local currency, thereby increasing the final retail and end-user cost.

Perhaps, a better way to conceptualize WTP is to align the easily accessible “black market” forex rate with the local currency value, which determines the purchasing power of the end user. This approach will likely give a more modest WTP estimate. Similar to the study by Durosinmi-Etti et al.,^1^ previous studies have also highlighted disparities in the amount willing to be paid for HIVST across country income settings, population types, and types of specimen collected (blood versus saliva).^8^^–^^14^ Respondents in middle-to high-income countries were more willing to pay a higher price for test kits (US$1.00 to >US$50.00) when compared to respondents from low-income countries (US$0.54–US$4.20) ^8^^–^^13^ while young unemployed individuals were willing to pay less for HIVST.^14^

Policy prescriptions by Durosinmi-Etti et al.,^1^ such as tax waivers on product importation, will go a long way to reduce the final cost of commodities to the end user. In addition, ongoing global price negotiation efforts for the prevention tools should factor exchange rate fluctuations in sub-Saharan Africa and strike multiyear fixed price deals. The final landing cost is more important for private providers and end users in low- and middle-income countries because supply chain and freight costs are very high in this global economic crisis. The ex works price includes the value of all the materials used and all other costs related to its production, minus any internal taxes, which are, or may be, repaid when the product obtained is exported. In ex works arrangements, the seller/manufacturer delivers the product to a designated location (usually at the port of the receiving country), and the buyer incurs transport costs and any damage to the product from the port to the point of delivery in-country. These additional in-country costs, largely from transportation, add up to the final landing cost if they are not factored in the initial contracts and negotiation with the seller/manufacturer.

Program managers working on HIV prevention should work with private provider networks to cap profit margins when selling to end users. Failure to do this could frustrate all market-shaping efforts and perpetuate barriers to access, affordability, and coverage. The ultimate goal is to achieve local production of these commodities, and this will require government dedication, policy support, and investment. The high WTP values of US$4.20 in the current study and US$5.20 in the previous study in Nigeria are concerning when there have been intensified global efforts to drive down HIVST prices and make testing affordable for end users. Presenting an inflated WTP without considering some of the issues previously mentioned may work against these global efforts, leading to keeping manufacturers’ prices high, minimizing their access by end users, and undermining the global public health effort to make HIVST accessible and affordable for all.

## Notes

**First Published Online:** January 25, 2023.
